# Multistage microsurgical reconstruction after catastrophic shark attack: Vascularized ulnar nerve grafting for a large segmental sciatic nerve defect

**DOI:** 10.1016/j.jpra.2026.02.016

**Published:** 2026-02-20

**Authors:** Jeremy Bishay, Harrison Garrett, Rowan Gillies, David Stewart, Bishoy Soliman

**Affiliations:** a Department of Plastic Surgery, Westmead Private Hospital, 12 Mons Road, Westmead, NSW 2145, Australia; b Department of Plastic Surgery, Royal North Shore Hospital, Reserve Road, St Leonards, NSW 2065, Australia

**Keywords:** Shark attack-related injuries (SARIs), Functional limb salvage, Peripheral nerve reconstruction, Traumatic nerve injury, Microsurgery, Vascularized nerve graft

## Abstract

We present a 48-year-old woman who sustained life threatening polytrauma from a shark attack in New Caledonia, including posterior trunk and thigh crush-avulsion, bilateral upper-limb amputations and a 16 cm segmental sciatic nerve defect. Staged reconstruction included a free latissimus dorsi (LD) flap to the posterior thigh, a vascularized ulnar nerve graft (VUNG) for sciatic repair, a neurotized anterolateral thigh (ALT) flap for sensate hand coverage, toe-to-thumb transfer and targeted muscle reinnervation (TMR). This approach achieved functional independence and quality of life restoration. This is the first reported use of a VUNG for large segmental sciatic nerve reconstruction.

## Background

Shark attack-related injuries (SARIs) can involve extensive soft-tissue avulsion, neurovascular injury and contamination, creating a hostile environment for reconstruction.^1^ In a retrospective review, Ballas et al. reported that 62% of survivors required amputation or disarticulation, with mortality rates approaching 38%, highlighting the severity of these injuries and the rarity of successful functional limb salvage.^2^

Definitive reconstruction typically requires staged, multidisciplinary management, yet existing literature largely focuses on debridement and amputation, with few reports of functional reconstruction.^1^^,^^2^

Vascularized Nerve Grafts (VNGs) have demonstrated improved axonal survival in long defects and ischemic or composite wounds. While vascularized sural grafts have been described for smaller sciatic lesions, VUNGs for large segmental lower-extremity defects have not been reported.^3^ This case describes a staged reconstruction using a VUNG to bridge a 16 cm sciatic defect in a contaminated, previously ischemic bed.

## Case report

### Initial presentation

**Figure 1:**
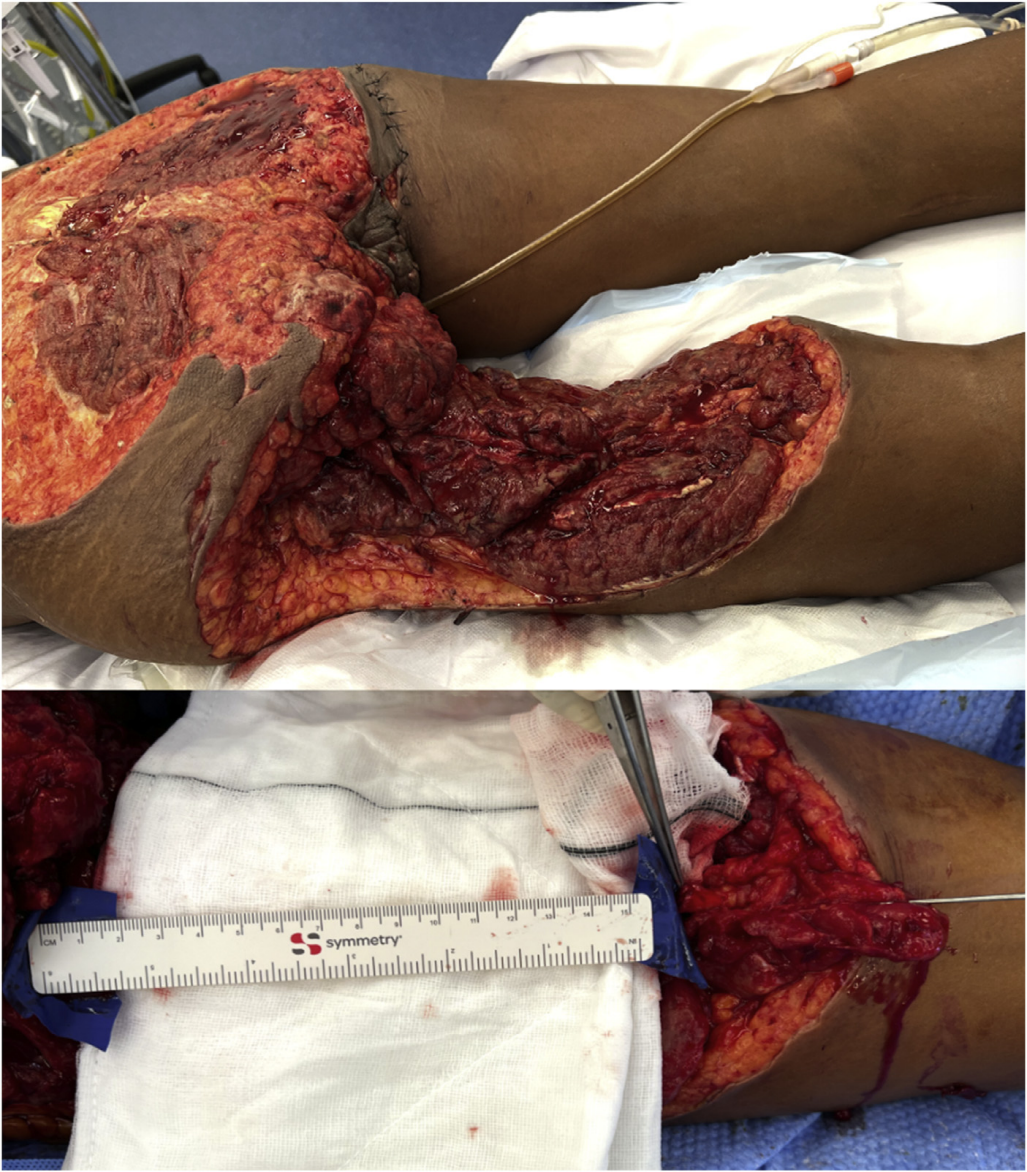
Initial Injury Pattern and Sciatic Nerve Defect – Clinical photograph showing an extensive composite soft-tissue defect of the left posterior thigh and buttock, with an adjacent image demonstrating a 16-cm segmental sciatic nerve defect measured intraoperatively.

A 48-year-old woman sustained catastrophic injuries following a shark attack in New Caledonia. After initial stabilization and colostomy diversion overseas, she was transferred for definitive reconstruction. Her injuries were extensive, including a 36 × 24 cm posterior trunk and left thigh defect with a 16 cm segmental sciatic nerve loss (Figure 1). The left hand was amputated at the level of the distal forearm, while the right hand had amputations of the thumb at the metacarpal head and of the index and middle fingers at the proximal phalanx. She also sustained an open left ankle fracture.

### Operative course

A multidisciplinary, staged reconstruction was performed over 6 months (February–July 2023), encompassing 7 major surgeries.

Stage 1 Initial stabilization and wound preparation

Serial debridements removed devitalized tissue and marine contamination. Revision amputations were undertaken for the non-viable left forearm and mutilated digits of the right hand. A bilaminar dermal substitute (BTM) was applied to the posterior trunk and left thigh.

Stage 2 Upper-limb reconstruction

Attention focused on the dominant right hand. A neurotized ALT free flap aimed to restore sensate coverage via coaptation of the lateral femoral cutaneous nerve to the superficial radial nerve and was anastomosed end-to-side to the radial artery. Subsequent flap revision improved flap contour.

Stage 3 Definitive lower-limb and sciatic nerve reconstruction

**Figure 2:**
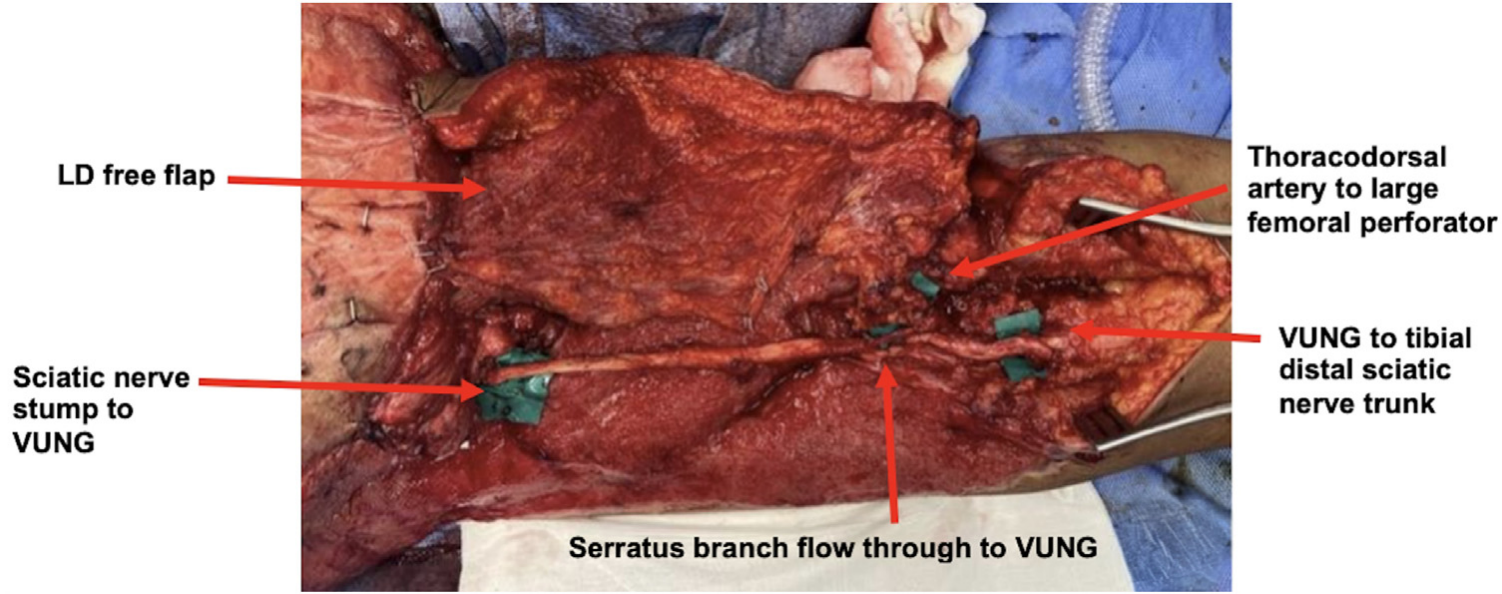
Lower-Limb Reconstruction Using a Flow-Through LD Flap and VUNG – Intraoperative photograph showing a flow-through LD free flap incorporating a VUNG via the thoracodorsal system to bridge a 16-cm sciatic nerve defect.

A free LD flap was harvested for coverage of the left posterior thigh defect. A 16 cm VUNG, based on the superior ulnar collateral artery (8 cm pedicle; 1.5 mm caliber), was used to bridge the sciatic defect. The ulnar nerve was selected due to its caliber match with the sciatic nerve, consistent vascular anatomy and the absence of the patients’ left hand following traumatic amputation. The VUNG was anastomosed to a serratus branch of the thoracodorsal system (2.5 mm) that was included with the LD flap as a flow-through construct with vascularity confirmed by brisk bleeding following saline irrigation (Video S1). Proximal coaptation was performed to the sciatic stump and distal coaptation to the tibial division, with the thoracodorsal artery anastomosed to a large femoral perforator (2.5 mm) (Figure 2). After flap inset, a split-thickness skin graft was applied to the LD muscle to complete coverage and refine contour.

### Functional reconstruction

**Figure 3:**
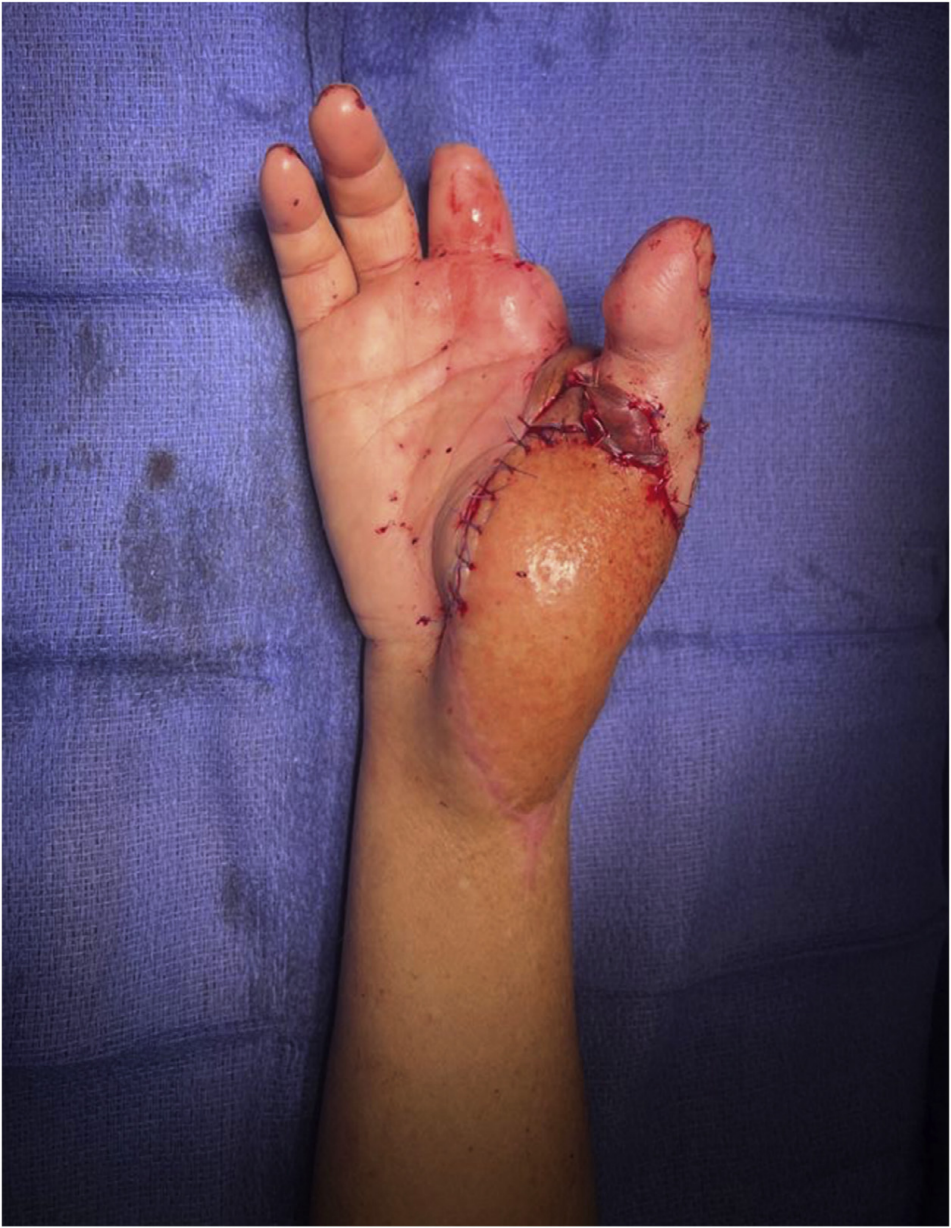
Thumb Reconstruction Using Great Toe Transfer – Postoperative clinical photograph showing thumb reconstruction using a great toe transfer following traumatic amputation.

A left great toe-to-right thumb transfer was performed using a first dorsal metatarsal artery pedicle, with 3 neural coaptations, 1 arterial and 2 venous anastomoses, tendon repairs and metacarpophalangeal arthrodesis (Figure 3). Although the Hallux was large, a trimmed toe transfer was not performed as the transfer was from the insensate left foot and therefore a trimmed toe would have excised tissue from what would become the more critical ulnar border of the thumb. TMR of the left forearm stump was performed (median > flexor carpi radialis; superficial radial > palmaris longus; proximal ulnar nerve > medial triceps) to reduce neuroma formation and neuropathic pain. Finally, left ankle arthrodesis was performed to prevent foot drop due to the segmental sciatic nerve injury, providing a stable platform for ambulation.

### Functional outcome

Rehabilitation between reconstructive procedures prioritized progressive weight-bearing, joint mobilization and prosthetic training. At 12 months following sciatic nerve reconstruction and ankle arthrodesis, the patient achieved independent ambulation with a single point stick. Knee flexion strength was graded MRC 4–5. Sensation is present in the plantar tibial nerve distribution. Following toe-to-thumb transfer, functional opposition and pinch were restored, with a Kapandji score of 7/10 and key pinch strength of 3.0 kg (approximately 55–60% of contralateral side). The neurotized ALT flap provided durable sensate coverage, with return of sensation and two-point discrimination of approximately 12–15 mm across the flap. Following TMR, there is no evidence of symptomatic neuroma.

**Figure 4:**
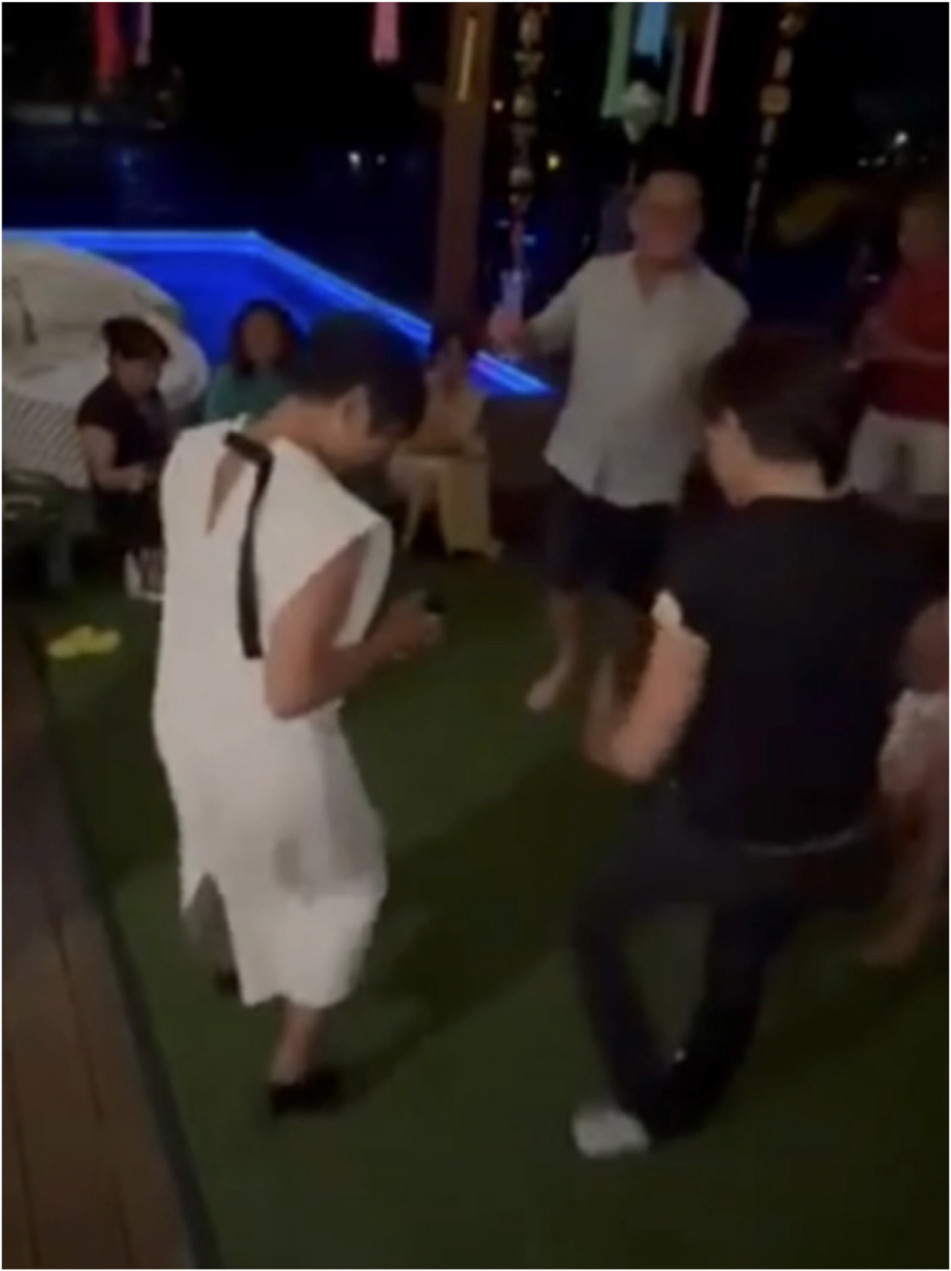
Advanced Functional Recovery at Long-Term Follow-Up – Clinical photograph showing independent dancing at long-term follow-up, reflecting restoration of balance, coordination and neuromuscular control.

She demonstrated fine control of a myoelectric prosthesis and electromyoprosthetic device on the contralateral side (Video S2). At 12 months, she was fully independent in activities of daily living, ambulating with a walking stick (Video S3) and participating in recreational activities including dancing and travel (Figure 4).

## Discussion

SARIs often cause multi-limb trauma with extensive soft tissue and nerve loss, positing major challenges to functional limb salvage.^4^ VNGs offer advantages over conventional grafts in long defects, ischemic beds and contaminated wounds.^5^^,^^6^ Although vascularized sural grafts have been used for smaller sciatic lesions, no published reports describe a VUNG for reconstruction of a large segmental sciatic defect. Doi et al. demonstrated superior axonal survival, electromyographic recovery and functional outcomes with VNGs compared with conventional grafts.^7^ Indications for VNGs include nerve gaps > 6 cm, non-vascularized recipient beds, composite defects requiring free tissue transfer, proximal lesions and prolonged denervation.^7^ In this patient, a 16 cm sciatic nerve gap in a previously avascular posterior thigh constituted an optimal indication for VUNG reconstruction.

This case also represents the first reported integration of multiple advanced microsurgical strategies in a single shark attack survivor – LD free flap reconstruction with a flow-through VUNG, neurotized ALT flap for sensate hand coverage, toe-to-thumb transfer and TMR. Although VUNGs are well described in upper limb reconstruction, their use in lower-extremity nerve repair is limited.^8^ In this context, the ulnar nerve represented an optimal donor, offering favorable caliber match and reliable vascularity without additional donor site morbidity due to the ipsilateral traumatic amputation.^9^ The flow-through configuration enabled simultaneous soft-tissue coverage and nerve reconstruction, maintaining intraneural perfusion across an ischemic, contaminated bed and supporting durable bridging of the sciatic defect with progressive clinical sensory recovery.

Toe-to-thumb transfer restored essential opposition and pinch, with the great toe providing optimal bone length, tendon alignment and neurovascular caliber while minimizing donor-site morbidity. TMR was employed to reduce neuroma formation and alleviate neuropathic pain. Careful donor-recipient selection maximized pain control without compromising residual motor function, demonstrating TMR’s dual role in functional rehabilitation and pain mitigation.

Formal electrophysiological assessment (EMG) of sciatic nerve regeneration was not performed due to the staged nature of reconstruction, socioeconomic factors and significant geographic barriers to follow-up. Despite this, meaningful neurological and functional recovery were demonstrated, including independent ambulation with a walking stick, restoration of plantar sensation, functional grasp and dexterity in the reconstructed right hand and effective use of a myoelectric prosthesis. Given the clear clinical improvement, EMG was unlikely to provide additional management-altering information. Objective functional measures, including Kapandji score, key pinch strength and two-point discrimination within the ALT flap, supported assessment of outcome. Several limitations should be acknowledged. As a single-case report with concurrent reconstructive procedures, the findings are not generalizable and attribution of functional recovery to any single intervention is limited.

## Conclusion

This case demonstrates successful staged reconstruction of a catastrophic shark attack, achieving meaningful functional recovery. The use of a VUNG to bridge a large segmental sciatic defect highlights the feasibility and durability of VUNG in lower-limb reconstruction, particularly in the unique context of a redundant ulnar nerve from a concomitant upper-limb amputation.

## Statements and declarations

We confirm we have read, understood, and complied with the JPRAS Open Guide for Authors, including all ethical, authorship, and reporting requirements.

## Data availability statement

Data sharing not applicable to this article as no datasets were generated or analyzed during the current study.

## Consent to participate

Written informed consent was obtained from the participant included in this study.

## Consent to publish

Written informed consent for publication of their clinical details and clinical images was obtained from the patient. A copy of the consent form is available for review by the Editor of this journal.

## Funding

None.

## Author contributions

All authors contributed to the study conception and design. The first draft of the manuscript was written by the first author and all authors commented on previous versions of the manuscript. All authors read and approved the final manuscript. Jeremy Bishay, MS, MD, BMedSt – writing original draft, editing and reviewing. Harrison Garrett, MS, MD, BMedSc – editing and reviewing. Rowan Gillies, FRACS (Plastic Surgery), MBBS – supervision, editing and reviewing. David Stewart, FRACS (Plastic Surgery), MRCS (Glas), MBChB – supervision, editing and reviewing. Bish Soliman, FRACS (Plastic Surgery), MS (Plastic Surgery), MBBS (Hons 1) – supervision, editing and reviewing.

## Declaration of competing interest

None declared.

## Appendix Supplementary materials

**Video S1.** VUNG bleeding following saline irrigation. Image, video 1 dummy alt text

**Video S2.** Patient flipping the cup on a table. Image, video 2 dummy alt text

**Video S3.** Patient walking independently with a walking stick. Image, video 3 dummy alt text
